# Scaffold-based tissue engineering strategies for soft–hard interface regeneration

**DOI:** 10.1093/rb/rbac091

**Published:** 2022-11-12

**Authors:** Yibo Li, Mo Zhou, Wenzhuo Zheng, Jianying Yang, Nan Jiang

**Affiliations:** State Key Laboratory of Oral Diseases, National Clinical Research Center for Oral Disease, West China Hospital of Stomatology, Sichuan University, Chengdu, China; State Key Laboratory of Oral Diseases, National Clinical Research Center for Oral Disease, West China Hospital of Stomatology, Sichuan University, Chengdu, China; State Key Laboratory of Oral Diseases, National Clinical Research Center for Oral Disease, West China Hospital of Stomatology, Sichuan University, Chengdu, China; Department of Outpatient nursing, West China Second University Hospital & Key Laboratory of Birth Defects and Related Diseases of Women and Children (Sichuan University), Ministry of Education, Sichuan University, Chengdu, China; State Key Laboratory of Oral Diseases, National Clinical Research Center for Oral Disease, West China Hospital of Stomatology, Sichuan University, Chengdu, China

**Keywords:** enthesis, tissue engineering, interface, mechanical properties, biomaterials

## Abstract

Repairing injured tendon or ligament attachments to bones (enthesis) remains costly and challenging. Despite superb surgical management, the disorganized enthesis newly formed after surgery accounts for high recurrence rates after operations. Tissue engineering offers efficient alternatives to promote healing and regeneration of the specialized enthesis tissue. Load-transmitting functions thus can be restored with appropriate biomaterials and engineering strategies. Interestingly, recent studies have focused more on microstructure especially the arrangement of fibers since Rossetti successfully demonstrated the variability of fiber underspecific external force. In this review, we provide an important update on the current strategies for scaffold-based tissue engineering of enthesis when natural structure and properties are equally emphasized. We firstly described compositions, structures and features of natural enthesis with their special mechanical properties highlighted. Stimuli for growth, development and healing of enthesis widely used in popular strategies are systematically summarized. We discuss the fabrication of engineering scaffolds from the aspects of biomaterials, techniques and design strategies and comprehensively evaluate the advantages and disadvantages of each strategy. At last, this review pinpoints the remaining challenges and research directions to make breakthroughs in further studies.

## Graphical abstract

**Figure:**
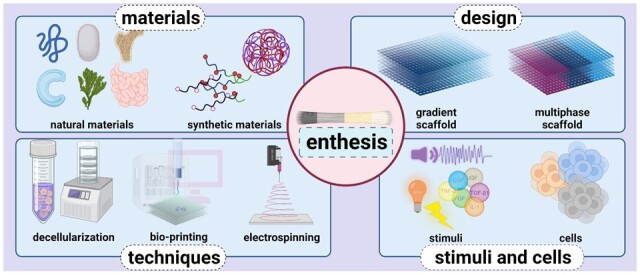

## Introduction

Movements, as the synergistic effect of the muscle–bone–joint system, partly depend on interfaces at one end of specific connections such as ligaments and tendons to obtain flexibility and stability. The soft–hard interface (enthesis) is a highly specialized site where tendon, ligament or joint capsule inserts into the bone and transmits tensile load from soft tissues to the hard tissue [1]. Therefore, they tend to become subject to sharp strain concentrations imputed to the sudden change in Poisson’s ratios, which should have separated the soft and hard tissues. Nevertheless, in adult patients, fractures of soft tissues like tendons and ligaments are likely to occur rather than the separation of soft and hard tissue, which is believed to be related to the gradient in composition and mechanical properties [2]. In particular, avulsion often occurs in adolescents, which may be caused by incomplete stress dispersion mechanisms [3, 4].

**Figure 1.**
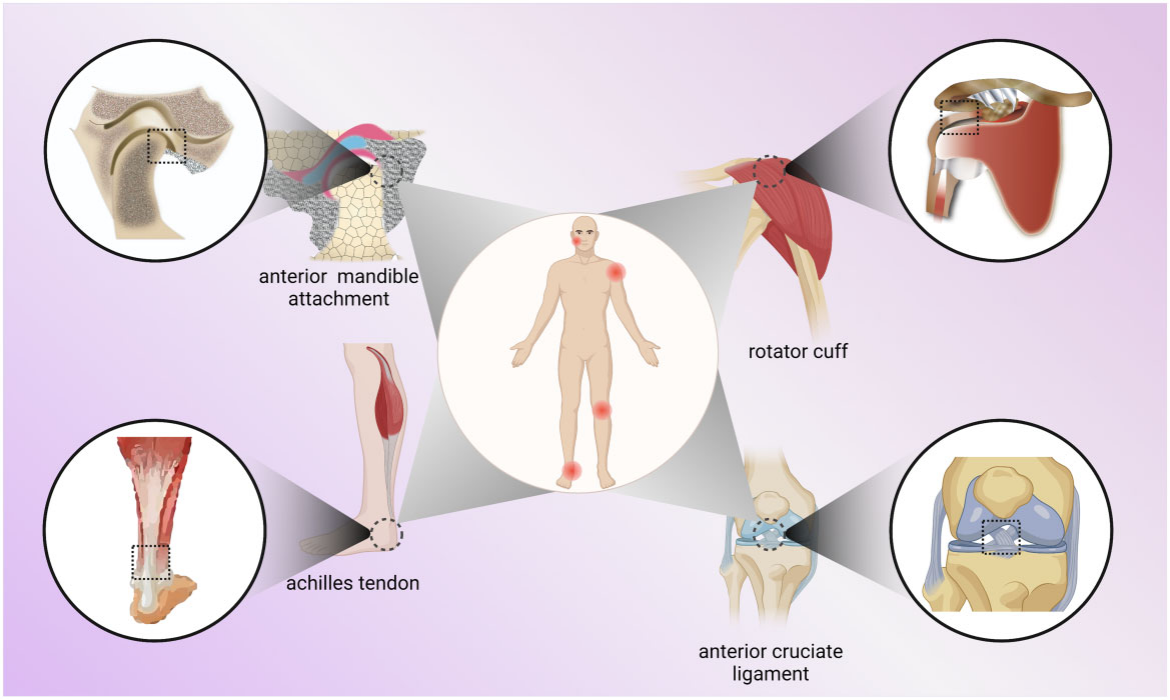
Examples of natural enthesis. Natural enthesis commonly consists of the tendon, uncalcified fibrocartilage, calcified fibrocartilage and bone. They exist in anterior mandible attachment, rotator cuff, achilles tendon, anterior cruciate ligament and some other soft–hard junctions.

**Figure 2.**
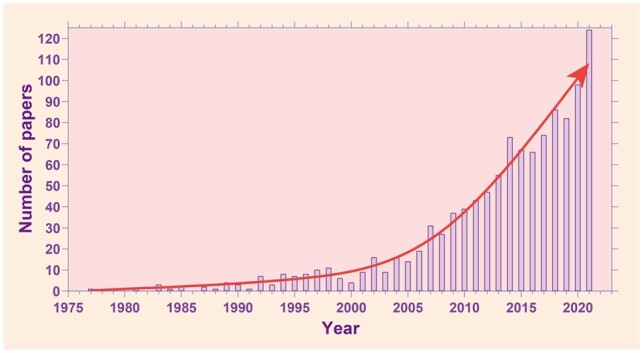
Evolution of enthesis research over the years. Data were retrieved from PubMed.org with the research designation ‘enthesis’, where 1000 results were found.

Enthesis injury can be triggered by trauma, vigorous force, failure of muscle coordination, post-traumatic arthritis, transitional exercise, aging, gender, weight, habits (such as smoking and sleeping), emotions etc [5]. The rotator cuffs, knee joints, ankles and temporomandibular joints are moved frequently or at a large angle and prone to soft tissue contusions, tears or breaks, resulting in restrictions on joint movement and seriously affecting the quality of life of patients (Fig. 1). In the USA, over 200 000 rotator cuffs are repaired and about 130 000 anterior cruciate ligament reconstruction operations are performed each year, resulting in a great economic burden [6]. Meanwhile, tendon or ligament injuries account for more than 30% of skeletal muscle diseases globally [7, 8]. To reestablish such structures, the soft tissue *in situ* is modified and fixed directly on the bone through the combination of anchors, sutures and bone tunnels, or the injury site is implanted with autologous or allogeneic tissue patches. However, movements are highly limited during recovery and the highly disorganized scar tissues located at the interface vary dramatically in mechanical properties, which may account for 20–95% recurrence rates after surgical repair [9]. Moreover, specialized enthesis tissue exhibits high precision in the mineral organization, cell types, fiber transition and biomechanical features at a nanoscale, and all these properties are significant for maintaining specific functions. Consequently, poor quality of repair frequently occurs, and more severe damage causes devastating results. It is shown in Fig. 2 that this area is getting much attention and is being increasingly explored. Among 1000 results, 104 papers are about repairing or regenerating enthesis via tissue engineering strategy because tissue engineering strategies can greatly improve postoperative healing success rates in current studies.

Tissue engineering is a tissue regeneration strategy integrating scaffolds, cells and stimuli in bioreactors. Implanting a regenerated engineering enthesis into the injured site can not only shorten the recovery time but also promote the tissue self-healing and restore the mechanical properties of the original structure (Table 1). Therefore, increasing studies focus on the exploration of biomaterials, techniques, stem cells, grown factors and mechanical stimuli to improve enthesis engineering.

**Table 1.** Comparison among natural enthesis, regenerative enthesis and scar tissues

	Compositions and structure	Mechanical properties	Ref.
Natural enthesis	Natural gradient tissue be divided into five parts: pure dense fibrous tissues (collagen I), uncalcified fibrocartilage (collagen II), tidemark, calcified fibrocartilage and bone tissue. The content of calcium rises from 0% (unmineralized region) to 60% (gradient region), then declines from 70% to 60% in the mineralized region.	Transfer loads effectively. Strain about 7% in the compliant region under 50 GPa.	[10–14]
Regenerative enthesis	Form gradient mineralization site in the middle with high cell viability. Achieve graded tenogenic and osteogenic differentiation along the mineral gradient.	Have better mechanical properties when they overcome the difficulty of layer-to-layer integration.	[15]
Scar tissues	Disorganized fibrovascular scar tissue consists of collagen III and gets gradually replaced with collagen I without collagen II. Lack of gradient mineral and collagen fiber distribution. Lack of undifferentiated cells. Reduction of cellularity and synthetic activity.	Excessive mechanical loading on the healing tendon. The mechanical strength is much lower, resulting in the high failure rate.	[10, 16, 17]

Some reviews have summarized the natural structure and functions of enthesis tissue [8, 9], factors affecting the development or healing of specific organs [3, 10–17], and criteria of engineering enthesis [5, 18]. Recently, increasing attention has been paid to growth factors (GFs) and their delivery [7], techniques to construct scaffolds [19, 20] and strategies combining scaffolds, cells and factors [21–23]. Nevertheless, we lack some perspectives that combine natural structure, properties and engineering strategies. Few reviews specifically concentrate on scaffold constructing methods, even though it is significant in mechanical property restoration and is the most difficult part of enthesis regeneration. Furthermore, the lack of a thorough summary about challenges and future perspectives limits new researchers from devoting themselves to this field. This review firstly focuses on the composition, microstructure, necessary bioactive cues as well as mechanical properties of several important entheses. Subsequently, the research progress of scaffold-based tissue engineering soft–hard enthesis is summarized, while promising biomaterials, advanced techniques, and creative designs. Finally, the current barriers and future perspectives in this field are discussed.

## Enthesis

### Compositions, structures and features of enthesis

**Figure 3.**
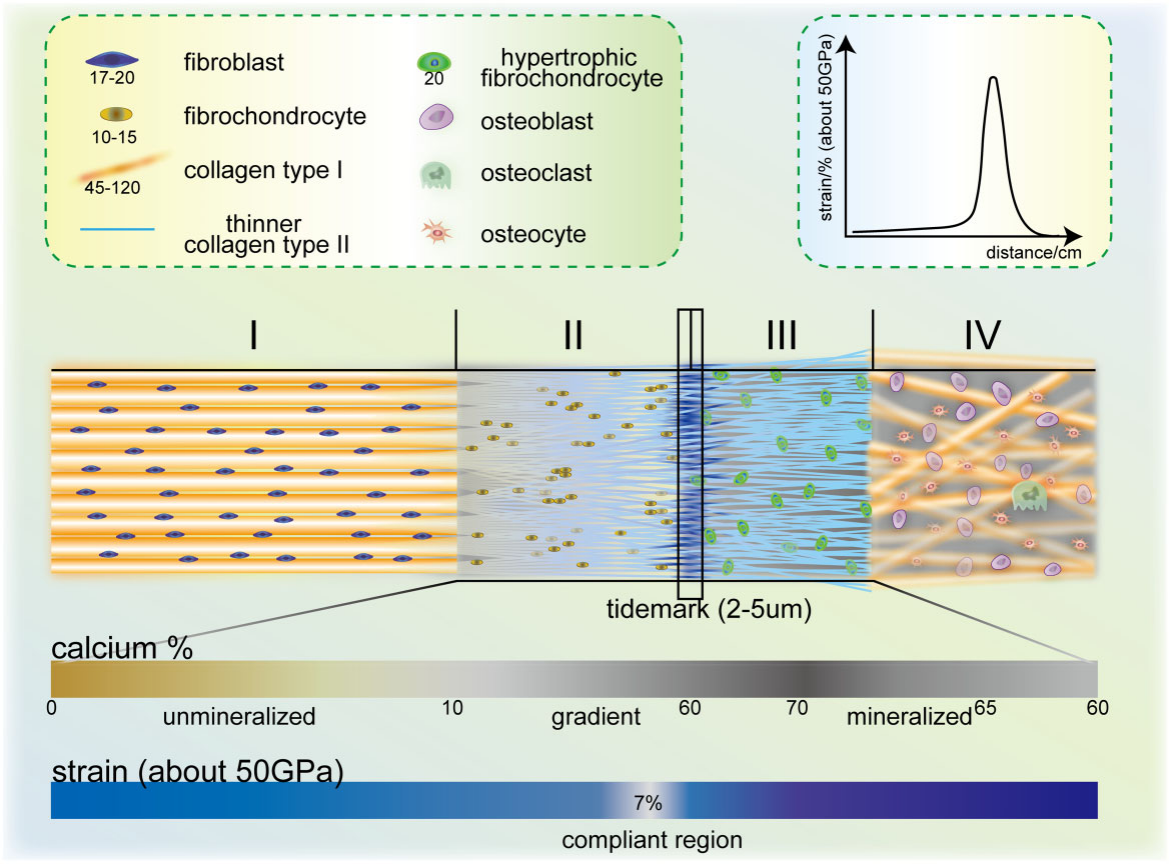
Compositions and structures of the natural enthesis. Structures of natural enthesis can be divided into five parts: pure dense fibrous tissues (I) including fibroblast (17–20 nm, 20–30 nm in other studies, long axis) and collagen type I (45–120, 20–200 or 50–200 nm in other studies, usually gathering into bundles). Uncalcified fibrocartilage (II) includes fibrochondrocytes (10–15 nm, long axis) and thinner collagen type II. Tidemark (2–5 μm). Calcified fibrocartilage (III) includes hypertrophic fibrochondrocyte (20 nm, long axis). Bone (IV) includes osteoblast, osteoclast and osteocyte. The content of calcium rises from 0% (unmineralized region) to 60% (gradient region), then it declines from 70% to 60% in the mineralized region. The strain is about 7% in the compliant region under 50 GPa [13].

According to the composition, enthesis can be further classified into fibrous and fibrocartilaginous enthesis. Dense fibers commonly exist in tendons or ligaments entering metaphyses or diaphysis of long bones, such as the deltoid tendon in the humerus, and they are directly attached to the bone in fibrous enthesis via the periosteum [10, 18]. Correspondingly, in epiphyses and apophyses, fibrocartilaginous enthesis could be roughly described as four transitional zones in early research (Fig. 3) [12, 19]. These four zones vary in collagen, minerals, cells and other substances as entheses stretch from soft to hard regions, for transferring external loads from soft tissues to bone, minimizing stress concentrations and facilitating joint movements. Pure dense fibrous connective tissue (zone I) mainly consists of highly aligned type I collagen and interspersed elongated fibroblast-like cells in the proteoglycans and glycoproteins matrix, where the low level of type III collagen and elastin can also be observed. Uncalcified fibrocartilage (zone II) is an avascular zone mainly consisting of type II collagen and type III collagen, which form a network-like structure for fibrochondrocytes [18]. Small amount of type I collagen and aggrecan are also present in this zone [10]. Specifically, following the uncalcified fibrocartilage is a basophilic line known as tidemark that separates zone II and III [14]. Calcified fibrocartilage (zone III) is also an avascular zone with type II collagen and hypertrophic fibrochondrocytes taking the predominant part and proteoglycan type I and X collagen dispersing around. Unlike the tidemark, this area is very irregular representing the junction between soft tissues and bone [10, 14]. The bone (zone IV) consists of osteoclasts, osteocytes, osteoblasts and mineralized type I collagen. Nevertheless, the von Kossa stain that used to observed and delimitate the four sections is lack of sensitivity in distinguishing densely mineralized regions from sparsely mineralized regions, resulting in limitation of the theory presented above and therefore requiring more accurate descriptions on multiple levels, which will be introduced later in this review [20]. Both fibrous entheses and fibrocartilaginous entheses exist extensively with crucial functions in human body. Here, given that fibrocartilaginous entheses are the fundamental part in transmitting tensile load and coordinating synergistic movements among muscle, bone and joint, also, relevant diseases have long been proved universal and challenging, we focus on the enthesis in this review.

Although some studies have proved that the presence of parallel type I collagen was just one of the stress minimization methods in line with the structures and components of entheses mentioned above [10, 14, 21] (the randomness has been proved to be a feature leading to smooth stress distribution [22]), such rough zoning of compositions cannot prevent critical stress peaks at the enthesis. Therefore, further observation of the composition and microstructure of the enthesis via microscopic technology is of great significance. At the leading edge of the hard–soft tissue interfaces (∼20 µm), combining transmission electron microscopy (TEM), X-ray micro-computed tomography, histomorphometry and Raman microprobe spectroscopy, a gradient in mineral was observed, which was related to the intrinsic surface roughness according to TEM imaging [20]. Nevertheless, a gradient increase existed in mineral content, which separated mineralized and unmineralized regions [23]. Other studies using TEM and Raman microscope found tendon inserts into bone over a disordered, smooth (or sharp) fibrocartilaginous transition, and the roughness changed with the length scale of observation [24]. At both the highest and the lowest length scales, the tissue and transitions appear ordered and smooth [20, 24]. However, at the intermediate mesoscale (∼100 nm to ∼10 µm), stochastic material distributions have emerged [24]. These differences are prone to result from bioapatite pockets, which cause the appearance of smooth grading at the nanometer scale [20]. In the transversal direction, a broadened attachment over ∼500 µm with thinner (diameter (13 ± 4) μm) enthesis fibers was proved, with high level of type II collagen and little type I collagen [22]. Furthermore, recent study showed there are differences of the cellular and molecular compositions of enthesis according to specific anatomical locations. For instance, the Achilles enthesis showed a more abrupt transition of soft–hard interface than other insertion site [25]. The further understanding of the interconnection of the collagen fiber structure with the mineralization network at the insertion site is of great significance to effectively designing biomimetic enthesis.

### Mechanical properties of enthesis

Effective transfer of loads is the most important function of the soft–hard enthesis. This structure should behave like a strong glue to connect two dramatically dissimilar materials, especially in tensile modules and stiffness. Take the insertion between bone (tensile modulus, ∼20 GPa; stiffness, 20 Gpa) and tendon (tensile modules, ∼0.45 GPa; stiffness, 200 MPa) as an example [26–28], the increasing risk of failure and stress singularities results from the mismatch. Despite the common sense that gradient concentrations of mineralization and protein reduce localized strains, other mechanisms also exist. One of the strategies is to form a highly compliant region, which has been observed by preparing samples via cryo-focused ion beam and then testing them via a special atomic force microscopy system [13]. Deymier declared that this zone may absorb energy and act as a crucial part in stress transfer, though it seems that this special zone is even more likely to cause catastrophic failure [13, 22]. Moreover, a higher level of deformation in the enthesis than in the fully unmineralized regions indicates that the modules affecting by mineral content are not the only factor contributing to mechanical properties [21, 29], and the compliance might be closely related to collagen orientation previously proposed [13, 21, 29]. In addition, considerably higher toughness and resilience were demonstrated on micrometer scale than that on millimeter scale in the same study. And this may result from the upregulation of fibril-forming collagens, fibril-associated collagens, and proteoglycans at the compliant region, which was observed via tandem mass spectrometry [22].

Another scientific hypothesis to reduce stress concentrations is the networked structure formed by enthesis fibers. A broadened attachment owing to the interdigitation of thinner fibers increases the area for load transfer, making a reduction of localized stress [22]. Moreover, given that rotational motions of joints often change the angle of stress quickly, the transverse mechanical response has been observed under different directions of stress. Interestingly, obvious heterogeneity occurred in the transverse direction, which was described as a recruitment of different groups fibers at the molecular level [30], especially when remote forces were applied.

Specifically, mechanical loading plays an important role during enthesis healing [31, 32], generation cellular gradient [33] and maturation [34], while the in-depth mechanisms including how such stimuli affect enthesis and how such special properties are generated need to be further explored.

### Stimuli for growth, development and healing of enthesis

Bioactive stimuli are vital for cell proliferation and tissue formation in soft–hard enthesis when they are growing, developing and healing. Appropriate stimulations can change mechanical properties of scaffolds when promoting the regeneration of bone and fiber as well as the accumulation of minerals. To make soft–hard enthesis functionally and morphologically similar to soft–hard enthesis microstructures, the significance of elements in soft–hard repair, including physical stimuli, chemical stimuli factors and biological elements should be paid attention to.

#### Physical stimuli

Physical stimuli, including ultraviolet ray, temperature, acoustic wave, mechanics, electric current and magnetic field, have a great impact on the formation of scaffold structure and the expression of bioactivity. When constructing scaffolds, physical crosslinking methods such as sintering and diffusion are effective for the connection and integration of various scaffold materials with specific structures formed through slicing and carving [15, 35]. As for other physical stimuli such as thaw-freeze cycles, voltage and mechanical forces, they commonly help form channels or micro-scale fiber structures in biomaterials, therefore providing sufficient space for cells to proliferate and differentiate.

Particularly, mechanical stimuli, including ultraviolet ray, acoustic wave (like ultrasound) etc. facilitate cell proliferation, differentiation and osteogenesis via stimulating biological cascades, increasing the level of GFs and cytokines, regulating gene expression and other ways. For example, above, level of vascular endothelial growth factor (VEGF) was improved by low intensity pulsed ultrasound (LIPUS), which led to the significant improvement of enthesis vascularity.

Interestingly, external force itself can reduce stress at the insertion site, too, which may be caused by one of the properties of the enthesis, anisotropy. Another research in 2019 presented an anisotropic viscoelasticity model, pointing out a protective mechanism mainly depending on viscoelasticity and reducing stress at higher strains [36]. Via an experimental observation with calculation the research found a mechanical response of the enthesis that under a biaxial tension setting, the loading stress decreases as the strain rate increase, while with a uniaxial tension set, the stress increases in strain rate, indicating a potential protective mechanism. This mechanism is essentially caused by the anisotropic nature of the enthesis, which is activated under biaxial tension in the experiment. Since few similarities were found in monodirectional strains, the situation of biaxial tension above is deemed to be closer to the physiological conditions of complex multiaxial loading.

Except for mechanical stimuli mentioned above, traditional physical stimuli such as electric treatment, ice pack, compression also have influence on the construction of enthesis applying tissue engineering. For instance, electric treatment plays an important role in enthesis tissue engineering. Specifically, the technique electrospinning is extensively used in fabricating nano-fibrous scaffold. With voltage applied, electrostatic charges accumulate and cause instability of polymer-solvent mixture droplets, and when repulsive forces overcome the surface tension of the droplet, the strand formation and movement are initiated [37]. Besides, the ice pack as a tradition method of stimuli, has the potential to facilitate the reconstruction of enthesis. What’s more, several studies demonstrated the role of compression in enhancing formation and mechanical properties of enthesis microstructures. When applied an 8% confined compression at the microscopic level of a polycaprolactone–collagen hybrid scaffold, a large part of cells attached to the collagen substrate were induced to form fibrous tissue [38]. Also, dynamic compression on biomimetic scaffolds is proved to be capable of enhancing the chondrocyte biosynthesis and matrix accumulation, suggesting the latent capacity for the use of cartilage tissue engineering [39].

These suggest that physical stimulation has a broad space for exploration as an important part of tissue engineering in soft–hard enthesis.

#### Chemical stimuli

Chemical stimuli have a wide range of use in soft–hard tissue engineering. Except for natural or polymer materials, which can facilitate the growth of cells and the formation of tissues mentioned before, common chemical stimuli include PH, ions, gases, bioactive molecules, minerals, enzymes, etc. Since the scaffold needs to be inoculated with cells and to simulate the microenvironment *in vivo*, most studies tend to focus on the consistence of chemical factors in the scaffold and the physiological state or cell culture environment.

To be specific, some chemical elements and compounds made up, as adhesives themselves, have been proved effective in osteoconduction and the construction of a biocompatible microenvironment at the insertion site, which facilitates cell proliferation and GF recruitment [40]. Injectable calcium–phosphate (Ca-P) matrix is helpful in forming soft–hard entheses that are structurally similar to protogenic soft–hard tissues with transforming growth factor (TGF)-β3 as well as the protogenic soft–hard tissues without TGF-β3. Similarly, some animal model studies pointed out that magnesium-based bone adhesives can enhance soft–hard formation through augmenting bone ingrowth toward the scar tissues [41, 42]. Moreover, a study showed the combination of f thin polyelectrolyte multilayers made of hyaluronan and chitosan with metal ions is helpful in the adhesion and differentiation of stem cells that support the construction of scaffolds. Specifically, the process is realized via ionic interaction, especially Ca^2+^ and Fe^3+^ with carboxylic groups of hydroxyapatite (HA). Also, low quantities of ions, for instance, impact greatly on cell behavior [43]. Additionally, low concentration of (PO_4_)^3−^ can cause the degradation of the scaffold structure [44].

Some bioactive molecules also have the function of augmenting the formation of soft–hard interfaces via stimulating the growth of different kinds of cells. An arginine–glycine–aspartic acid coating can enhance tenocyte proliferation, while a collagen coating of sutures can stimulate adhesion as well as protein synthesis. Additionally, gelatin methacryloyl (GelMA), a promising biomaterial for engineering scaffolds and drug carriers, and Kartogenin (KGN), are combined to create better effects. KGN can facilitate selective differentiation of bone marrow mesenchymal stem cells into chondrocytes [45]. Together they help form soft–hard interfaces and improve the mechanical properties [45].

Moreover, studies have indicated traditional Chinese medicine, as a new kind of chemical stimuli, could promote soft–hard interface regeneration. Specifically, the proliferation of osteoblast, such as MC3T3-E1, is promoted by icariin via enhanced expression of bone morphogenetic proteins (BMPs) including BMP-2, BMP-6, BMP-7 and biglycan [46]. Also, icariin promotes the mineralization with icariin-loaded bioactive scaffold by upregulating the expression of alkaline phosphatase (ALP), osteocalcin and osteopontin [47, 48]. Additionally, traditional Chinese medicines are of great activities of anti-bacterial, anti-apoptotic and antioxidant, which might contribute to the formation of soft–hard interfaces [49].

Chemical stimuli are continuously discovered and tested. When constructing soft–hard interfaces and scaffolds, flexible and guarded use is of significance.

#### Biological stimuli

Under the impacts of endogenous and exogenous factors, stem cells can differentiate in various directions. Due to this property, stem cells are studied in tendon-to-bone scaffold construction in order to form an area similar to protogenic insertion site via the process of cell proliferation and differentiation. Several animal model studies have proved the effectiveness of stem cell application in improving tendon-to-bone healing [40]. However, except for some inherent expressions, stem cells depend on GFs to help the formation of enthesis, while most GFs extensively exist in the human body and act through various signaling pathways and cascades. For instance, it has been discovered that the factor Alpha-smooth muscle actin (a-SMA) is expressed in early soft–hard attachments, contributing to the tunnel integration in the formation of enthesis.

GFs could be divided into stimulatory and inhibitory factors when used in tendon-to-bone scaffold construction (Table 2). Specifically, several osteoinductive GFs: TGF, BMP, fibroblast growth factor (FGF), platelet-derived growth factor (PDGF) and granulocyte colony-stimulating factor can be used as stimulatory factors and are most intensively and deeply studied. These GFs have positive and significant effects on the reconstruction of soft and hard tissues [40, 50]. Major inhibitory factors in soft–hard construction include matrix metalloproteinases (MMPs) and tumor necrosis factor-α (TNF-α). MMPs are enzymes that are involved in the degradation of the extracellular matrix (ECM) that forms the connective material between cells and adjacent tissues, while TNF-α is a potent inflammatory mediator, stimulating osteoclast activity and thus inhibiting osteoblast differentiation [51]. They both delay the healing of soft–hard entheses.

**Table 2.** Growth factors and their main sources, functions involved in construction of entheses

Factor		Effects	Main sources in natural forming process	Ref.
Stimulatory factors	TGF	Promote the proliferation and differentiation of mesenchymal derived cells, especially the proliferation of osteoblasts and fibroblasts Promote the expression of ECM Inhibit the growth of osteoclasts Participates in regulation of the homeostasis and remodel of the enthesis	Platelets; bone extracellular matrix	[40, 56]
	BMPs	Members of TGF family (except for BMP-1), promote the proliferation of osteoblasts and fibroblasts, as well as the formation of ECM	Osteoprogenitor cells; chondrocytes; osteoblasts; osteoclasts	[40, 56]
	FGF	Stimulate the proliferation of fibroblasts and help form the matrix	Chondrocytes; macrophages; osteoblasts	[40, 56]
	PDGF	Promote the proliferation of fibroblasts, glial cells and smooth muscle cells	Platelets; macrophages; osteoblasts	[40, 56]
	VEGF	Stimulate the growth of vascular endothelial cells, help form blood vessels in entheses and thus help maintain blood supply at the enthesis	Chondrocytes; osteoblasts; mesenchymal cell; endotheliocyte	[57]
	IGF	Promote the growth of various cells of enthuses	Chondrocytes; osteoblasts; MSCs	[57]
	GDF	Promote the growth and differentiation of various cells of entheses	MSCs; chondrocytes;	[57]
	G-CSF	Stimulate the formation of the enthesis; intensively studied	Immune cells; osteoblasts	[40, 56]
	α-SMA	Promote tunnel integration in formation of entheses	Myofibroblasts	[40]
Inhibitory factors	MMP	Breakdown the connective material between cells and adjacent tissues through degrading the extracellular matrix	Fibroblasts; leukocytes	[51]
	TNF-α	Stimulate osteoclast activity and inhibit osteoblast differentiation	Macrophages; immue cells	[51]

Platelet-rich plasma (PRP), as an autologous derivative of whole blood, which contains considerably rich cytokines including GFs, TGF, insulin-like growth factor-1, PDGF, FGF, VEGF and interleukins. In this proper environment, different kinds of cells more actively proliferate and migrate *in vitro* and in animal studies compared with controls [52]. Specifically, one of the main components of PRP, PDGF, has an impact on the mitogenic effects on osteoblasts, and tenocytes, promoting the healing of soft–hard tissue [9, 53].

The loading approaches of GFs are of great significance since effective delivery and release capabilities ensure the functions of various GFs. Several strategies have been reported in recent years. In a recent study on loading methods of GFs, researchers achieved controlled delivery of efficiently loaded GFs via layer-by-layer (LBL) assembly with ∼90% efficiency in the stimulation of appropriate pH, which increased the ionic strength as well as the gas plasma surface activation. The LBL assembly also provided a sustainable controlled release of different GFs, making use of the interaction of polyelectrolytes as well as biological binding and substrate surface traits [54]. Moreover, De Witte *et al*. [55] came up with degradable poly (methyl methacrylate)-co-methacrylic acid nanoparticles to deliver GFs to targeted sides without losing bioactivity. They incorporated GF proteins into nanoparticles whose degradation rate was controlled by tuning the number of hydrolytically ester units, protecting GFs from inactivation and enabling their sustained release. The high affinity between the nanoparticle and BMP-2 was proved, too [55].

Those bioactive stimuli mentioned above could work in numerous complex networks with different factors interacting with each other. It is possible and prospective for us to explore and conclude different pathways which could be applied in the design and construction of structured scaffolds.

## Scaffold-based tissue engineering strategies

Scaffolds play the role of simulating ECM in tissue engineering. The ideal scaffold is consistent with the physiological enthesis in morphology and function through precise design and proper manufacturing. Given the complexity of biochemical composition and microstructure at the enthesis, scaffolds in this field should meet these criteria: Macroscopically, the scaffolds should be designed considering different organs and individuals as well as differences between physiological and pathological tissue in morphology; the sudden change of tensile modulus at the enthesis requires high strength of biomaterials; effective adhesion to soft connective tissues and bones is the key for *in vivo* functions; scaffolds’ degradation rate and new tissue development are synchronized; low toxicity should be certified. At the micro-scale, scaffold materials should possess appropriate porosity to meet the space and gas needs for cells and tissue growth; the channels should be specially reserved to satisfy the delivery or transportation of GFs, cell secretions, cells, fibers to create a multi-cell-factor system; in particular, the microstructure heterogeneity of both transverse and longitudinal sides of the enthesis can reduce the stress concentration, so scaffolds also need to simulate this heterogeneity or improve the integration between layers to bear the increased stress. To meet the above requirements, the materials, techniques and designs of scaffolds should be carefully decided (Fig. 2).

### Biomaterials of scaffolds

The materials of enthesis regeneration include natural materials and synthetic materials (Table 3). To date, natural materials such as fibrin, collagen, chitosan, elastin, hyaluronic acid, silk fibroin, acellular matrix and bioactive inorganic materials have been considered. Notably, Chinese tradition medicine also are very useful as materials or stimuli in repairing the enthesis. For example, icariin, resveratrol and epigallocatechin promote chondrogenesis by upregulating the expression of SOX9, AGG and college I [58]. The unified advantage of these materials is their excellent biocompatibility, while most of them have poor mechanical properties and rapid degradation and shrinkage, especially when they are not well-processed. Solving the problem of poor biomechanical properties, synthetic materials have higher strength but also limitations in coexisting with active cells. Polylactic acid (PLA) [59], poly-L-lactic acid (PLLA) [60], polyglycolic acid (PGA) [61], poly (lactide-co-glycolic acid) (PLGA) [42, 62, 63], polyethylene glycol (PEG) [35], Poly desaminotyrosyl-tyrosine ethyl ester (PDTE) [64], Polycaprolactone (PCL) [65–67] and so on, have been used to design some functional parts of the scaffolds.

**Table 3.** Common biomaterials used in enthesis engineering and brief summary of their advantages and disadvantages

	Acronym/chemical formula	Extended name	Advantages	Disadvantages	Ref.
Natural protein	SF	Silk fibroin	Biodegradability Mechanical properties Possibility to promote fiber growth	Poor cell recruitment properties	[69, 79–84]
	Col	Collagen	Biocompatibility Biodegradability	Poor mechanical properties	[85, 86]
Bioactive inorganic materials	Ca_3_(PO_4_)_2_	calcium phosphate	Osteoconductivity		[35, 71]
	CPS	Ca_5_(PO_4_)_2_SiO_4_	Biocompatibility Biodegradability		[71]
	HA	Hydroxyapatite	Bioactivity Biocompatibility Osteoconductivity	Slow biodegradation	[66, 87–89]
Synthetic materials	PLLA	Poly-L-lactic acid	Biodegradability Mechanical properties Easily processed		[72, 90]
	PLGA	Poly (lactide-co-glycolic acid)	Possibility of surface functionalization Biodegradability Mechanical properties Possibility to promote fiber growth		[85, 91]
	PGA	Polyglycolic acid	Possibility of surface functionalization Biodegradability Mechanical properties		[92]
	PCL	Polycaprolactone	Porosity Biodegradability Easily processed Biocompatibility Possibility to promote tissue formation and mineralization	Low bioactivity Quick biodegradation	[65, 67, 87, 89, 90, 93, 94]

The advantages of natural protein represented by fiber, collagen and silk fibroin are to promote fiber growth in tissue *in vivo* or in tissue engineering models, and to integrate soft and hard tissue. Notably, xenogeneic silk fibroin can not only promote fiber regeneration and cell viability at the fracture but also has better mechanical properties than collagen. The seemingly smooth surface of silk fibroin fibers is actually composed of many oriented nanofiber bundles, which have a fishnet-like structure composed of β-pleated sheet and amorphous chains formed by nano-fibril. The β-sheet structure acts as a stress-bearing node, while the amorphous chains connect each node (β-sheet structure) like a rope, forming a flexible and strong structure [68]. Of note, many scientists dissolve silk fibroin and then recast it to obtain regenerated silk fibroin with better mechanical properties, which indicates that the complex multidimensional structure is the result of self-assembly. Chen’s team constructed a layer-by-layer scaffold using nanofilament silk fibroin, which performed well in the subsequent growth and differentiation of bone marrow mesenchymal stem cells, ultimate load-to-failure and chondrogenesis [69]. *In vivo*, gradient mineral silk fibroin scaffold has successfully loaded a pullout force of 10.61 ± 0.83N, which was significantly stronger than the control group. The wide application of silk protein encourages us to explore more natural materials for tissue engineering.

Bioactive inorganic materials, such as calcium phosphate, calcium hydroxy phosphate, calcium phosphate silicate (CPS) Ca_5_(PO_4_)_2_SiO_4_, etc., can generate the mineral gradient at the enthesis. Graded mineralization of the four layers is one of the key factors of function. To be specific, the atomic molar ratio of calcium and phosphorus in HA is 1.67, which is very close to the inorganic component of enthesis tissue [70]. HA is often compounded with other materials, thereby endowing bioactivity to the scaffold to promote cell growth and tissue generation. Zhao *et al*. [71] compared the cell adhesion and osteogenesis of CPS, HA and non-materials in the repair process, and found that CPS and HA had obvious advantages, among which CPS showed better biocompatibility and biodegradability. The fabrication of organic/inorganic scaffolds such as fibrous membrane containing HA and PLLA [72] or PCL [73] showed greater load strength and improved collagen/cell growth. Interestingly, contrary to the strategy of compounding polymers and bioactive ceramics/glass, some studies achieve the transition between soft and hard tissue through gradient demineralization [74, 75].

PCL is gradually recognized as a scaffold material because it is free of toxicity and acidity of degradation products while it promotes tissue formation and mineralization. By a combination of specific techniques, PCL can be made into nanofibers to form anisotropic or aligned-random fiber arrangements. Wang *et al*. [76] significantly increased the breaking stress and achieved sustained drug released (maintain 60% freshly prepared materials, 2 months) by preparing a core-shell structure of PCL/silk fibroin heavy chain (H-fibroin) (75:25). Some layer-by-layer scaffolds simulated the different calcification of tendons, fibrocartilage and bone by changing the ratio of PCL to tricalcium phosphate (TCP) [77], hydroxyapatite (HAp) [66] and so on. Shuang’s team compared the promotion of tissue regeneration by aligned PCL (aPCL), nonaligned PCL (nPCL), aPCL-collagen I, nPCL-collagen II and nPCL-nanohydroxyapatite scaffolds *in vivo*, demonstrating the possibility of early implantation and reconstruction of composite materials [67]. These reflected its potential as a part of synthetic materials.

Furthermore, the composite of some molecules with scaffold materials can significantly improve the performance of scaffolds. Font Tellado *et al*. [78] compounded heparin on a biphasic silk fibroin scaffold to increase the ability of cells to produce TGF-β2 and GDF5. The KGN-loaded Gelatin methacryloyl (GelMA) hydrogel scaffolds showed better mechanical properties and increased new cartilage tissue [45]. Song *et al*. [65] loaded melatonin on PCL membrane, which increased the regeneration of cartilage-like tissue and collagen when partially reduced vascularization.

Given the special functions of the enthesis to connect soft and hard tissues, biomaterials to regenerate bone, cartilage and dense connective tissue often construct parts of a scaffold. In addition, since scaffolds are often multi-layered, adhesive materials may serve as functional parameters in the regeneration of enthesis tissues. Ideal materials’ mechanical performance should change gradually with the position to prevent sudden increases of stress concentration caused by sudden changes of Young’s modulus.

### Advanced techniques applied to scaffolds

**Figure 4.**
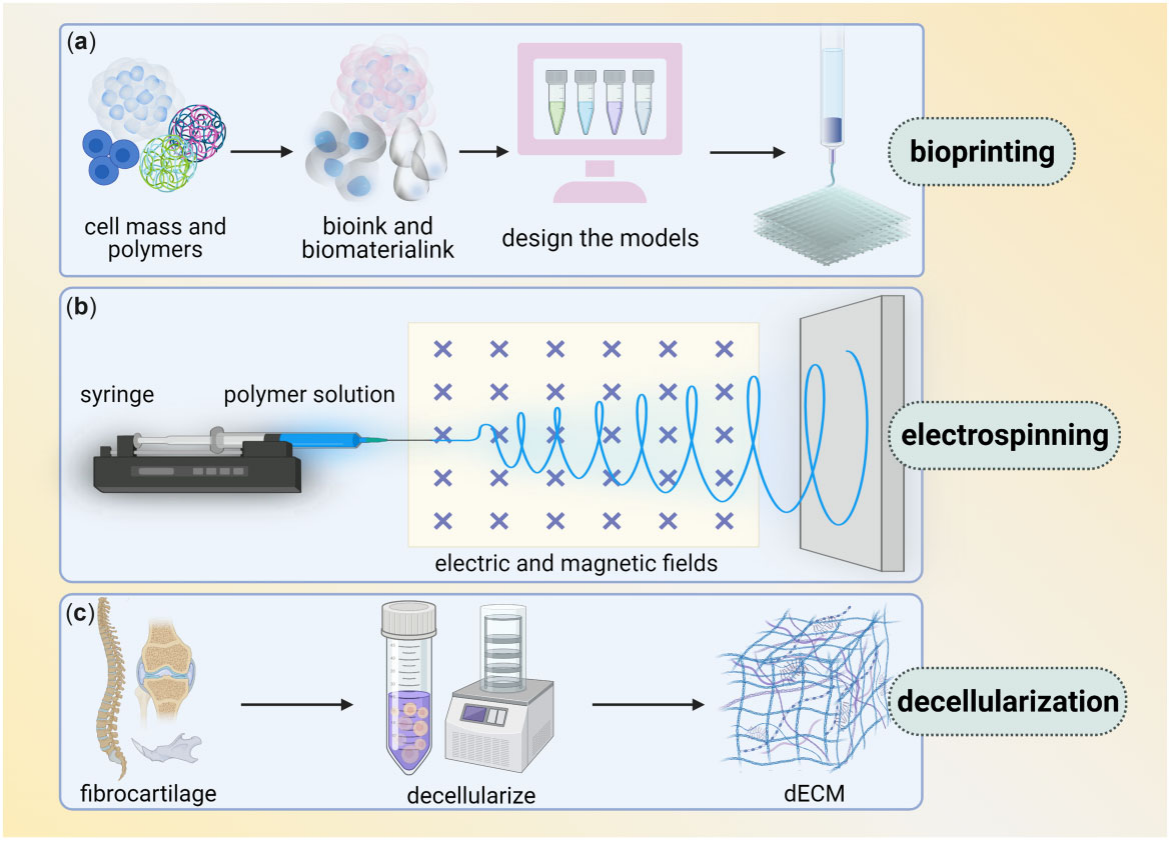
Main techniques of enthesis tissue engineering (several techniques are commonly used in enthesis engineering, including bioprinting, electrospinning and decellularization. (**a**) Bioprinting, in which bioink and biomaterial ink are extracted from cell mass and polymers, then models are designed and printed layer by layer. (**b**) Electrospinning use syringe to let polymer solution pass through electric and magnetic fields. (**c**) Decellularization transfers fibrocartilage tissue into dECM).

With the limitation of the low accuracy of traditional self-assembly technique and thermally induced phase separation technology, advanced scaffold fabrication techniques such as LBL self-assembly, electrospinning, bioprinting, decellularization make it possible to reconstruct complex gradient structures of enthesis tissues (Fig. 4). Meanwhile, some techniques are applied for sustained release, one example is that GFs were encapsulated in PLGA microspheres by a double emulsion [95].

#### Bioprinting

3D bioprinting is to accurately obtain target tissues, organs or scaffolds based on models designed by computer using biocompatible biomaterial inks or biological inks containing cells. Though limited by the biomaterials and the change of mechanical properties of scaffolds after printing, 3D bio-printing is still one of the best choices in fabricating scaffolds. 3D printing can not only make specific scaffolds with high precision and speed but also meet individual demands and reduce rejection. In enthesis tissue engineering, bioprinting is often used to construct multiphase scaffolds with complex structures and functions, when most of the 3D printed scaffolds are layer by layer. And biomaterial inks are processed to attain specific properties beforehand. Generally, mechanical features come from drying, multiple freeze-thaw cycles and infiltrating different materials, while infiltrating of GFs, surface modification and other helper techniques contribute to an initial bioreactor. Cao *et al*. [77] printed three-phase scaffolds in a layer-by-layer fashion from tendon (PCL, two layers) and fibrocartilage (PCL/TCP, four layers) to bone (PCL/TCP, three layers), and bone marrow mesenchymal stem cells and osteoblasts were encapsulated in GelMA and equipped on them. Tarafder *et al*. [93] printed a three-phase scaffold to allow micro-precise spatiotemporal delivery of GFs. However, 3D printed scaffolds are limited to temporarily retaining morphology and microstructure *in vitro*, which indicates that the technique needs to be further improved.

Interestingly, 4D printing adds the time dimension than 3D printing, paying more attention to accuracy and the changes of the scaffold over time under stimulations. This statement seems to be perfectly in line with the tissue engineering because implanted materials will degrade in the internal environment. Due to the relative stability of the internal environment, using 4D printing to accurately deliver factors, slow-release drugs, and maintain mechanical properties is promising. Another possible improvement is the application of bio-inks, which consist of bioactive materials, living cells, biocrosslinkers and stimulating factors. The successful reconstruction of a high-fidelity auricle shows the possibility of using bio-inks for complex enthesis engineering [96].

#### Electrospinning

In electrospinning, polymers are dissolved in organic solvent when high voltages are applied. The electrostatic charge overcomes the surface tension of droplets above some specific voltage threshold to form a stable jet [97]. Often, the stream loads positive charges on the surface and runs toward negative electrode [98]. When the charged jet is ejected under electric field, the solvent evaporates to narrow and form a submicron fiber scaffold. To achieve specific form of insertion, researchers often concentrate much on the diameter and arrangement of fibers, which can be designed by controlling several parameters, which are classified as solution parameters, process parameters and environmental parameters [99]. How these parameters affect the appearance and properties of fibers have been discussed in detail before [100]. In enthesis fabrication, most importantly, a time-dependent process and complex crosslinking of materials are needed to make the scaffolds gradient and have specific mechanical properties. Cong *et al*. [67] used electrospun PCL scaffolds to cross-link different types of collagen layer by layer, while Nowlin *et al*. [101] focused on asymmetric fiber arrangement to construct random-to-aligned nanofiber scaffolds. Different from the two, Zhao *et al*. [62] used emulsion electrospinning technique to fabricate ultrafine fibers with a core-sheath structure to maintain bFGF activity, and constructed bFGF-loaded electrospun poly (lactide-co-glycolide) fibrous membranes, which induced more collagen tissue regeneration and maintained the strength *in vivo*. These studies show that electrospinning can help integrate ultrafine nanofibers, customize fiber arrangement, design core-shell structure and so on, so that the tissue regeneration strategies can go to the nanometer scale.

Even though electrospinning is widely used, fiber adhesion and thickening caused by low concentration is also one of the main reasons for its failure. Furthermore, similar to 3D printing, poor fiber strength limits the durability of mechanical properties of scaffolds. An ideal strategy is to encapsulate bioactive substances and signaling molecules within the electrospun fibers to facilitate the interaction between scaffolds and cells.

#### Decellularization

Given that biological scaffolds take over the role of natural ECM, removing the cellular components from ECM is a good strategy for the construction of scaffolds. The decellularization technique arose, producing decellularized extracellular matrix (dECM) retains the biochemical composition and mechanical properties of natural tissues, as well as excellent biocompatibility. dECM can be obtained by physical, chemical or biological treatments of bones, cartilages, dermis, small intestinal mucosa, tendons, ligaments and enthesis tissues [102–104]. Physical methods such as multiple freeze-thaw cycles, stirring and negative pressure intend to destroy the cells’ structures and their attachments with the matrix; cutting, punching, physical crushing, etc. are to improve the efficiency of decellularization, especially when they are applied in combination with other methods; in addition, when combined with other technologies such as laser processing or three-dimensional printing, decellularization is more promising to generate the biological scaffolds in shape and function. Chemical methods use chemical reagents to destroy cells, when retaining the composition and reducing drug residues is necessary. Biological methods use nucleases, collagenases and other substances to specifically remove cells’ crosslinks. To keep a balance that removes the cellular components and retains the matrix, above techniques are often not used alone. For example, Su applied a technique that combined physical, chemical, and enzymatic treatments to build a decellularized bone-fibrocartilage-tendon (D-BFT) composite scaffold [105]. dECM can be directly used as a scaffold when cells are seeded and factors are integrated, and it can also be implanted *in vivo* for *in situ* regeneration.

More often, these substrates are reprocessed into composites and into specific shapes. Derda stacked the paper-supported hydrogel-matrix layer by layer, resulting in the gradient change of oxygen concentration, which provided the possibility for the three-dimensional cell culture system [106]. Olvera *et al*. [107] found that tissue-specific ECM L-ECM and C-ECM (microfilaments formed by electrospinning) can specifically induce tissue formation, then added HA to the distal end of C-ECM to promote bone formation. More choices for obtaining ECM and standards of dECM for various tissue engineering need to be further studied.

### Design of structured scaffolds

At present, two general strategies for designing the enthesis are to generate the enthesis alone or to regenerate it as a part of bone/ligament/tendon engineering (Table 4). For example, some ligament tissue engineering models could bear the increased stress of compliant zone and achieve soft–hard connection by changing the arrangement of fibers [108] or the type of materials [109] at ends. This review focuses on generating a separate enthesis.

**Table 4.** List of construction strategies of engineering enthesis

Biomaterials	Fabrication technique	Stimulus	Design	Scaffolds used in different organs and animals	Results	Ref.
PGA-PLCL	Electrospinning	Glycolic acid	Creation of microporous structures with fiber diameters within the nanometer range	Rotator cuff; sheep	Display an enthesis similar to protogenetic insertion without surgical complications	[92]
hDCB-ECM	Decellularization	Chemokine (SDF-1)	Graded demineralization for gradient scaffold	Rotator cuff; rabbit model	Promote stromal cell recruitment, bone and fibrocartilage formation and ultimate tensile stress	[74]
PCL/HA/ZnO	Electrospinning	1% (ITS) phalloidin	Composite films with less bacterial attachment	*In vitro*	Promote cell compatibility ,adhesion, osteogenesis chondrogenesis and fibrocartilage formation	[87]
CGCaP/PEG/CG	Lyophilization; cross-linking	None	Continuous triphasic scaffolds containing osseous and tendinous tissue compartments	*In vitro*	Dissipate interfacial strain between mechanically disparate tissue compartments	[35]
PCL–PCL/TCP–PCL/TCP	3D printing	Loading	Three phases; different cells were separately encapsulated in GelMA and loaded seriatim on the relevant phases of the scaffold	Rotator cuff; mice	Promote cell seeding, chondrogenesis, and matrix deposition in varying phases	[77]
Tissue-specific ECM; AP	Electrospinning	ECM-derived components	Multiphasic scaffold; distal region of the C-ECM coated fibers additionally functionalized with an apatite layer	*In vitro*	Promote MSC differentiation, cartilage template development toward different tissues	[107]
ECM	Decellularization	HAp (mineral content)	Scaffolds with mineral gradients	*In vitro*	Cells in matrix with higher mineral content display osteogenic behavior at earlier times than those in the unmineralized substrate	[110]
Silk fiber	Electrospinning	SBF	A nanofibrous scaffold with gradient mineral coating	Anterior cruciate ligament; rats	Enhance integration in the tendon-to-bone enthesis with a higher ultimate load and more fibrocartilaginous tissue formation	[69]
ECM	Decellularization; freezing; section	SDF-1α	A scaffold with book-shaped structures with 5 pages (about 3 × 2.5 × 0.25 mm), page thickness = 50 μm)	Rotator cuff; rats	Avoid the complex process of *in vitro* loading cells on the scaffold and is convenient for clinical application	[111]
KGN; GelMA	Ultraviolet crosslinking; vacuum freeze-drying	Bone marrow stimulation	A KGN-loaded GelMA hydrogel scaffold	Rotator cuff; rabbits	Improve enthesis healing by promoting fibrocartilage formation and improving the mechanical properties	[45]
GelMA	3D printing	TGFβ family growth factors	A multi-phasic gelatin methacrylate hydrogel construct system for spatial presentation of proteins	*In vitro*	Guide heterogeneous and spatially confined changes in mesenchymal stem cell genes and protein expressions	[112]
HAp/PCL	Swelling	ASCs	A gradient scaffold patterned with an array of funnel-shaped channels	*In vitro*	Help form a functionally graded enthesis	[66]
PCL	Electrospinning	EM (electromagnetism)	Scaffolds contain nanofibers positioned in various direction	Rotator cuff; rats	Enhance early enthesis reconstruction	[67]
Trabecular bone	Demineralization	bfGF	A scaffold with an apatitic mineral gradient using a top-down approach	*In vitro*	Generate a model showing the dependence of mineral removal as function of time in the chelating solution and initial bone morphology	[75]
Melatonin; PCL	Electrospinning	TGF-b3, melatonin-PCL extracts	Melatonin-loaded aligned PCL electrospun fibrous membranes were fabricated	Rotator cuff; rats	Inhibit macrophage infiltration in the tendon-to-bone enthesis at the early healing phase, increase chondroid zone formation, promote collagen maturation, decrease fibrovascular tissue formation and improve the biomechanical strength of the regenerated enthesis	[65]
Silk fiber	[113]	None	[113]	Anterior cruciate ligament; sheep	Make ACL regeneration with a silk fiber-based scaffold with and without additional cell seeding	[84]
Titanium	3D printing	None	A lattice structure of 500 mm diamond unit cells, deep in the column and connected to the lattice on the top of the lateral surface of the ascending ramu	TMJ; patient	promote bone formation and reattach lateral pterygoid enthesis	[114]
PCL	3D printing	CTGF	Three-layered scaffolds with micro-precise spatiotemporal delivery of growth factors	Rotator cuff; rats	Show translational potential for improving outcomes after rotator cuff repair	[93]
PCL; PET	Layer-by-layer self-assembly	BMP-7 WAC	To roll up PCL nanofibrous membrane and PET mesh fabric into a ‘swiss roll’ structure	Anterior cruciate ligament; rabbits	Promote the integration of hybrid ligaments and bone tunnels	[94]

Taking the change of composition and mineralization gradient at the enthesis into consideration, the scaffold is generally designed as a multiphase rather than a single. Multiphase scaffolds have multiple functional layers with multiple compositions and performances, so they bear greater tension and provide microenvironments for multiple cells. Different materials or techniques are often used to simulate different layers which differ in structures and functions. Mosher *et al*. [95] constructed a three-phase scaffold for anterior cruciate ligament (ACL) to simulate bone, fibrocartilage and ligament, respectively, when cultured fibroblasts, chondrocytes and osteoblasts. Of note, the phases were closely jointed to form networks by sintering. Zhu *et al*. [15] designed a three-phase scaffold that integrates with tendon or bone at both ends and forms a gradient mineralization site in the middle. Particularly, uniaxially aligned channels (ACs) are constructed to accurately guide fiber arrangement and cell distribution. Above design strategies mainly cater to the layering of composition, mechanical properties and characteristics described in section ‘Compositions, structures and features of enthesis’.

In fact, the physiological enthesis is continuous, thus the continuously graded scaffolds focus on the micron (or nano) scale changes of mineral gradients and fibers, avoiding layer-to-layer mutation in multiphase scaffolds. One strategy is to coat calcium phosphate on or incorporate it into electrospun nanofibers [115]. Zhu *et al*. [15] coated varying concentrations of Hap on the top surface of opaline lattice infiltrated by layered PLGA to establish a controllable mineral gradient. In addition, the gradient can be made by transplanting osteogenic transcription factors Runx2/Cbfa1 into fibroblasts [116]. Boys *et al*. [75] achieved continuous mineralization through gradient demineralization of bone trabeculae. In subsequent osteogenic tests, cells exposed to more minerals showed earlier osteogenesis [110]. Cai *et al*. [83] designed a dual-layer aligned-random silk fibroin/P(LLA-CL) nanofibrous scaffold and proved that it is applicable *in vivo*, which shows the importance of layering and fiber arrangement. Chen *et al*. [117] engraved the electrospun aligned fibers to form surfaces’ roughness micro-and-nanometer. The continuous scaffolds usually have better mechanical properties when they overcome the difficulty of layer-to-layer integration.

The regenerated bone-tendon/ligament enthesis vary in structure and mechanical properties by organs, so it is necessary to discuss properties in different positions from a separate perspective. The most reported are enthesis at the end of rotator cuff, Achilles tendon and anterior cruciate ligament. Currently, 112 papers on [(‘Rotator Cuff’[Mesh]) AND (enthesis)], 94 papers on [(‘Achilles Tendon’ [Mesh]) AND (enthesis)] and 34 papers on [(‘Anterior Cruciate Ligament’[Mesh]) AND (enthesis)] are included in the NCBI database. Apparently, studies of the enthesis at the end of tendons superfluous ligamentous enthesis. Partly because tendon-bone enthesis tend to experience greater stress and rupture by overstretching, while ligaments maintain stability. The shearing between fibers components and the expansion of fibers are main mechanical effects when tendon stretching [118]. Execute behaviors of tendon fibers depend on relative mechanical properties of the fibers and surrounding matrix, which are difficult to quantify and vary with genetics, age, sex, diet, etc. For ligaments, mechanical properties are related to time and history. Before ligaments stretching to the failure point, the stress–strain curve has toe region, elastic region and plastic region. These three regions correspond to straightening of the crimped fibers, increased elastic loading and increased plastic loading with fibers’ starting to fail, respectively [119]. The above characteristics suggest that enthesis at the end of tendons needs to conduct shearing between fibers, while enthesis at ligament-bone junction needs to be closely monitored the dynamic changes of the fibers. However, few studies have specifically focused on the microscopic mechanical differences between tendons and ligaments. In fact, some *in vivo* experiments have demonstrated that poor mechanical behavior is a fatal problem for enthesis scaffold [69, 111, 120, 121].

We summarize the latest research or applications in each position (Table 5). A recent study reported a novel acellular rotator cuff scaffold. The researchers fabricated a book-shaped decellularized enthesis matrix (O-BDEM) with well-tensile property via a decellularization approach based on a vacuum aspiration device. As a result, the novel decellularization strategy shortened the time of decellularization from 16 to 7 h, while preserved more enthesis ECM (especially the minor matrix components) [122]. Some new materials, for instance, poly(ester-urethane) urea crimped nanofiber and nano-calcium silicate mineralized fish scale were applied in rotator cuff scaffold in 2022 [123, 124]. For the enthesis at Achilles tendon, the PCL-5%HA-1%ZnO films have been proved to have great application potential in repairing the bone-tendon interface. The promotion was inducted via the regeneration of the four layers structure of bone-tendon interface, including osteogenesis, chondrogenesis, fibrocartilage construction and tendon healing. Specifically, calcium deposition and collagen I and II expression were facilitated with increased ALP, BMP-2, RUNS2 and collagen type I alpha 1 chain (COL1A1) expression on PCL-5%HA-1%ZnO films. Furthermore, the film showed great antibacterial effects, reducing the infection in the process of enthesis healing [87]. As for anterior cruciate ligament (ACL), researchers reported an injectable, uniform and stable HA/collagen paste, which effectively promotes cell proliferation, osteogenic expression, ECM deposition and functional regeneration of the enthesis. To be detailed, the interconnected porous structure which was inferred the final porosity of around 40% could provide compositional and spatial support to the tissue regeneration in the bone tunnel and appropriately proliferated osteogenically express including high expression of ALP and PMP-2 (a kind of polysaccharides). And the cell number in the pastes increased significantly in the process of 14 days. All above lead to a better healing of the tendon-bone interface of the ACL [125]. In these studies, however, mechanical properties remain challenging. All regenerative scaffolds have disparity with natural tissues in terms of stiffness, viscoelasticity, fiber dynamics and so on. Some studies avoided mechanical testing of implants, possibly because the scaffold promotes fast healing of the enthesis and the stage to support function can be negligible.

**Table 5.** List of latest development of enthesis in tendons/ligaments

	Tendons	Ligaments	Ref.
Compositions and structure	Connective tissue mainly including collagen and specialized cells tenocytes. Collagen constitutes 65–80% of the dry mass of healthy tendons, including collagens types I, II, III, V, VIII, IX, X, XI, XII, XIV and XIX–XXI. The basal membrane is composed of type IV collagen.	Dense connective tissue consists of fibroblasts, fibrocartilage and mainly collagen type I or elastin. Directly or indirectly attached to the bone tissue, with the indirect attachment through Sharpey fibers.	[126, 127]
Mechanical properties	Different due to various elements including classification and methodological factors. The strongest tendon in human body is the Achilles tendon, with the ultimate stress of 100 MPa, and the ultimate strain of 4–10%. The attachment side of the soft and hard tissue is a weak point in deformation.	Different due to various elements including classification and methodological factors. The attachment side of the soft and hard tissue is a weak point in deformation.	[126, 127]
Development of enthesis in tendons/ligaments	Constructing scaffolds with appropriate biomaterials, hierarchical organized architecture and specially designed structures *in vitro*. Cultivation of cells and integration of factors on scaffolds. Enhancing mechanical properties and testing engineering enthesis *in vitro*. Performing *in vivo* implantation with histological evaluation but finding limitations in mechanical behavior.	Leveraging the advancements from biomaterials to replicate the native features of enthesis *in vitro*. Finding cells leading to accelerated healing. Improving mechanical properties including gradient in stiffness, shear loading and stress. Injecting cells/biomaterials in animal models, but without scaffold grafting.	[59, 122, 128–132]

## Challenges and future perspectives

**Figure 5.**
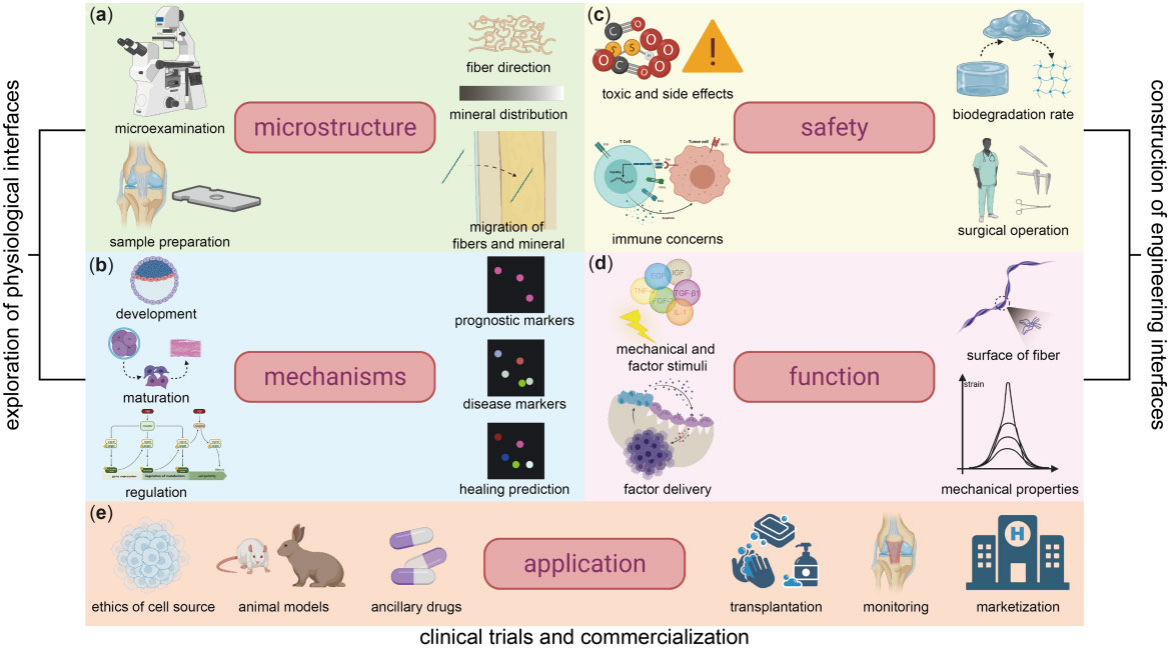
Challenges of enthesis and its regeneration (challenges within exploring physiological entheses are shown in (**a**) and (**b**). (**a**) Inadequate microscopic observation techniques, insufficient understanding of fiber direction, mineral distribution, complexity of fibers’ migration and obstacles in sample preparation. (**b**) Barriers in exploring mechanisms of embryogenesis, healing and regulation progress and few markers of three stages. Challenges within constructing engineering enthesis are shown in (**c**) and (**d**). (**c**) Obstacles in ensuring safety involving toxic and side effects, possible immune concerns, uncertain biodegradation rate *in vivo* and surgical operation risks. (**d**) Little knowledge of mechanical stimuli and factor stimuli, complex factor delivery, lack of techniques to edit the surface of fiber and mechanical properties of biomaterials. Challenges within clinical trials and commercialization are shown in (**e**). (**e**) Few insecure cell sources, incomplete animal model system and a lack of suitable ancillary drugs in clinical trials. (Lack of effective strategies for transplantation, postoperative monitoring and marketization of products.)

Despite the spring up of scaffold-based enthesis regeneration strategies, some challenges are still here to be overcome (Fig. 5). Firstly, further studies referring to the composition, structure and function of the enthesis are still needed, which require higher accuracy. In this sense, it may help make a breakthrough in this field to focus on the observation of fibers at the nanoscale via advanced microscopic devices and to refine sample preparation (Fig. 5a). Besides, the dynamic variation of fibers and minerals distribution under stress stimulus needs finding out (Fig. 5a). For example, Rossetti *et al*. [22] found the directional variability of fibers in the transverse direction when given different force actions, while it remains a mystery whether local minerals migrate under the mechanical stimulus and become unevenly distributed. Secondly, it is essential to make clear the embryogenesis, healing and regulation mechanisms of enthesis, which are expected to guide the construction of bioreactors involving various stimuli (Fig. 5b). Moreover, if researchers can find prognostic biomarkers of some diseases, early intervention may provide a better therapeutic effect (Fig. 5b). In short, a more sufficient understanding of the physiological enthesis is expected to provide guidance for constructing bionic engineering enthesis.

Moreover, a deeper and more accurate understanding of the composition, structure and function of the enthesis at different scales as well as the relevant mechanism is helpful to further elucidate how biomechanical properties in compliant region form, while optimism of engineering enthesis scaffolds techniques and stimulation strategies are vital to restoring certain mechanical properties. Practically, the focus of researchers in this field has also gone through several different stages as factors affecting mechanical properties are constantly being re-understood. At present, people seem to pay more attention to the improvement of mechanical properties by designing fibers’ arrangement, distribution and microstructure. However, how the randomness of fibers affects mechanical properties requires more detailed molecular studies and rich mechanical knowledge to elucidate. Furthermore, the smooth stress distribution is only an unclear understanding of the enthesis’s function, while dynamic changes in structure and transfer of substance under specific stress are likely to be more related to the mechanical properties. More efforts are needed to figure out mechanical properties of the enthesis and to reconstruct mechanical properties of them.

Although the development of materials and techniques endows the engineering enthesis scaffolds with biocompatibility and good mechanical properties, it is still necessary to optimize the methods to achieve the combination of function and safety. For materials, porosity and degradation rate *in vivo* should be paid special attention, because they are related to the living of cells, the efficiency of factors delivery and the maintenance of mechanical properties (Fig. 5c). Nevertheless, there are few methods to monitor the degradation rate *in vivo*, and consequently laboratory animals are often performed a second operation to observe the transplants. To cope with this, researchers can apply minimally invasive or noninvasive imaging techniques to manage laboratory animals.

Also, researchers in this field need to optimize current stimulation strategies and achieve abundant cellular interactions. More studies concerning the effects of the combination of stimulus intensity, time and type need to be conducted, especially the combination of various GFs (Fig. 5d). Of note, immunological rejection between cells is critical in the regeneration of enthesis. Accurately designing channels and microchambers in the scaffold will achieve multi-cell coexistence and immune concerns reduction.

Additionally, researchers need also seek more appropriate experimental animals to check the biocompatibility of tissue-engineered scaffolds and formulate standards for animal models in achillea tendon, temporomandibular joint and other soft to hard enthesis to facilitate clinical implementation (Fig. 5e). So far, animals that have been used to study the enthesis are almost always rabbits (especially in rotor cuff) and rats, for the rabbit rotator cuff has an open surgical field and these small animals are easier to raise. However, we need larger animals to build more models that resemble the human body. Going even further, the postoperative pain of patients needs closely watching with particular attention to inflammatory changes (such as osteoarthritis) and toxic degradation components (Fig. 5e). Besides, chronic diseases and recovery of movements also need long-term management. At present, scaffold-based implants have been clinically used in skin [133], meniscus [134], periodontal tissue [135, 136], etc. Some clinical trials had also been carried in bone [137, 138], cartilage [139], ligament [140], heart [141] and other tissues. These studies may provide strategies for clinical translation of regenerative enthesis. Considering the ethical concerns and possible immune rejection, cord blood bank cells and induced pluripotent stem cells may be ideal cell sources.

To summarize, the clinical translation and commercial use of engineering soft to hard enthesis still require extensive preclinical experiments and tests, but it is still an ideal strategy to improve current surgical treatments.

## Funding

This study was supported by grants from the National Natural Science Foundation of China (Grant/Award Number: 81901026) and the Department of Science and Technology of Sichuan Province (Grant/Award Number: 2021YFH0139).

*Conflicts of interest statement*. None declared.

## Compliance with ethics guidelines

This article does not contain any studies with human or animal subjects performed by any of the authors.
